# Endothelial dysfunction in acute ischemic stroke: a review

**DOI:** 10.1007/s00415-025-12888-6

**Published:** 2025-01-15

**Authors:** Antonia Kleeberg, Thomas Luft, Daniel Golkowski, Jan C. Purrucker

**Affiliations:** 1 Department of Neurology, Heidelberg University Hospital, Heidelberg, Germany; 2 Department of Oncology and Hematology, University Hospital Heidelberg, Heidelberg, Germany

**Keywords:** Endothelial dysfunction, Endotheliopathy, Acute ischemic stroke, Endothelium, Stroke

## Abstract

**Background and Purpose:**

Endothelial dysfunction is considered an emerging therapeutic target to prevent complications during acute stroke and to prevent recurrent stroke. This review aims to provide an overview of the current knowledge on endothelial dysfunction, outline the diagnostic methods used to measure it and highlight the drugs currently being investigated for the treatment of endothelial dysfunction in acute ischemic stroke.

**Methods:**

The PubMed® and ClinicalTrials.gov electronic databases were searched for eligible articles/studies dealing with endothelial dysfunction and stroke. The references of the articles were screened to identify additional sources. The data were abstracted and summarized.

**Findings and discussion:**

Endothelial dysfunction can be measured by serum biomarkers as well as by ultrasound or plethysmography techniques. Drugs targeting endothelial dysfunction include widely used agents such as angiotensin-converting enzyme inhibitors or isosorbide mononitrate, but also experimental therapies such as endothelial progenitor cells.

**Conclusion:**

The role of endothelial dysfunction in acute ischemic stroke has been studied increasingly in recent years. It has been shown that there is a correlation between endothelial dysfunction and parenchymal hematoma after endovascular thrombectomy. Also, early clinical trials are conducted investigating, e.g., endothelial progenitor cells in the treatment of endothelial dysfunction in ischemic stroke. Current research focuses on the integration of novel markers of endothelial dysfunction into routine clinical practice to support decision making in the treatment of acute ischemic stroke.

## Introduction

There is growing evidence that endothelial dysfunction exerts a crucial role in various cardiovascular diseases including ischemic stroke [1, 2]. The endothelium is known to convey several important functions beyond forming the barrier between blood vessels and the perivascular space. Together with the basal membrane, the pericytes and the astrocyte end feet, the capillary endothelium of the brain forms the blood–brain barrier (BBB) [3]. The endothelium regulates vascular tone by producing numerous vasoconstrictive (endothelin [ET], angiotensin II, Thromboxane A2, reactive oxygen species [ROS]) and vasodilative factors (nitric oxide [NO], prostaglandins, endothelium-derived hyperpolarizing factor [EDHF]). It regulates fibrinolysis and coagulation and plays a role in angiogenesis and inflammation [1, 4]. Notably, there is not one single endothelium, but endothelial cells differ in different tissues and vessel types by their structure (e.g. extent of intercellular junctions), function (e.g. in terms of permeability, leucocyte trafficking) and expression of serum biomarkers (such as adhesion molecules, anti-/procoagulant molecules), reflecting the differential function of different vessels. This is thought to be due to an interplay of microenvironment and (epi-)genetic modification [5, 6]. Endothelial cells differ from each other not only spatially but also temporally. Certain genes form a kind of mosaic pattern, which, according to current knowledge, is stochastically “switched” on and off in different endothelial cells of the same tissue over time [7].

Regarding the BBB, this capillary endothelium differs from other types of capillary endothelium. It possesses many tight junctions, a reduced number of caveolae (organelles that mediate transcytosis, the transfer of molecules across the endothelium) and therefore exhibits lower permeability, reflecting the barrier function of this particular endothelium [5, 8, 9].

This review aims to provide an overview of the current knowledge on endothelial dysfunction, outline the diagnostic methods used to measure it, and highlight the drugs currently being investigated for the treatment of endothelial dysfunction in acute ischemic stroke.

## Methods

**Fig. 1:**
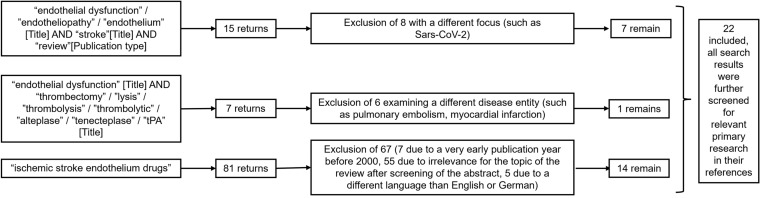
Flow diagram of included/excluded articles

To identify papers eligible for this review, PubMed® was searched. For a first broad overview, the search string “endothelial dysfunction”/”endotheliopathy”/”endothelium” [Title] AND “stroke”[Title] AND “review”[Publication type] was used to search for previously published reviews on this topic. This search yielded 15 results, eight of which were excluded due to a different focus. We also searched for “endothelial dysfunction” [Title] AND “thrombectomy”/”lysis”/”thrombolysis”/”thrombolytic”/”alteplase”/”tenecteplase”/”tPA” [Title]. This search yielded seven results, six of which were excluded because they referred to a different disease entity. As to possible therapies, a search for “ischemic stroke endothelium drugs” was performed without further specification. It yielded 81 results, 14 of which were considered further; the others were disqualified as irrelevant (see Fig. 1 for a flow diagram of included/excluded articles). ClinicalTrials.gov was searched for relevant studies on drugs for the treatment of endothelial dysfunction in stroke. All search results were further screened for relevant primary research in their references. Only English- or German-language manuscripts were considered further. The data were then abstracted and summarized.

## Findings and discussion

### Definition of endothelial dysfunction

When normal endothelial function is disrupted by external factors such as shear stress, excessive ROS formation or activation of the renin–angiotensin–aldosterone system (RAAS), the endothelium is unable to perform its normal functions and endothelial dysfunction occurs. Endothelial dysfunction is generally described as a condition in which vasodilation is impaired, inflammation, proliferation and fibrosis occur, and prothrombotic factors are present [1, 10]. Other sources define endothelial dysfunction as a condition in which the bioavailability of the vasodilative factors NO or EDHF is reduced [11, 12].

The mechanisms mediating these changes are heterogenous, including genetic predisposition, cardiovascular risk factors and oxidative and mechanical stress [10]. To illustrate, atherosclerosis causes inflammation in the perivascular space, e.g. through the formation of ROS or leakage in the endothelium, through which fluids and proteins enter the perivascular space [12]. This leads to a thickening of the arterial walls due to fibrosis, which impairs vasodilation and results in occlusion and thrombosis [13, 14]. In cerebral vessels, impaired vasodilation leads to impaired autoregulation and thus to reduced cerebral blood flow [15]. Moreover, the blood–brain barrier is disrupted [13].

**Fig. 2:**
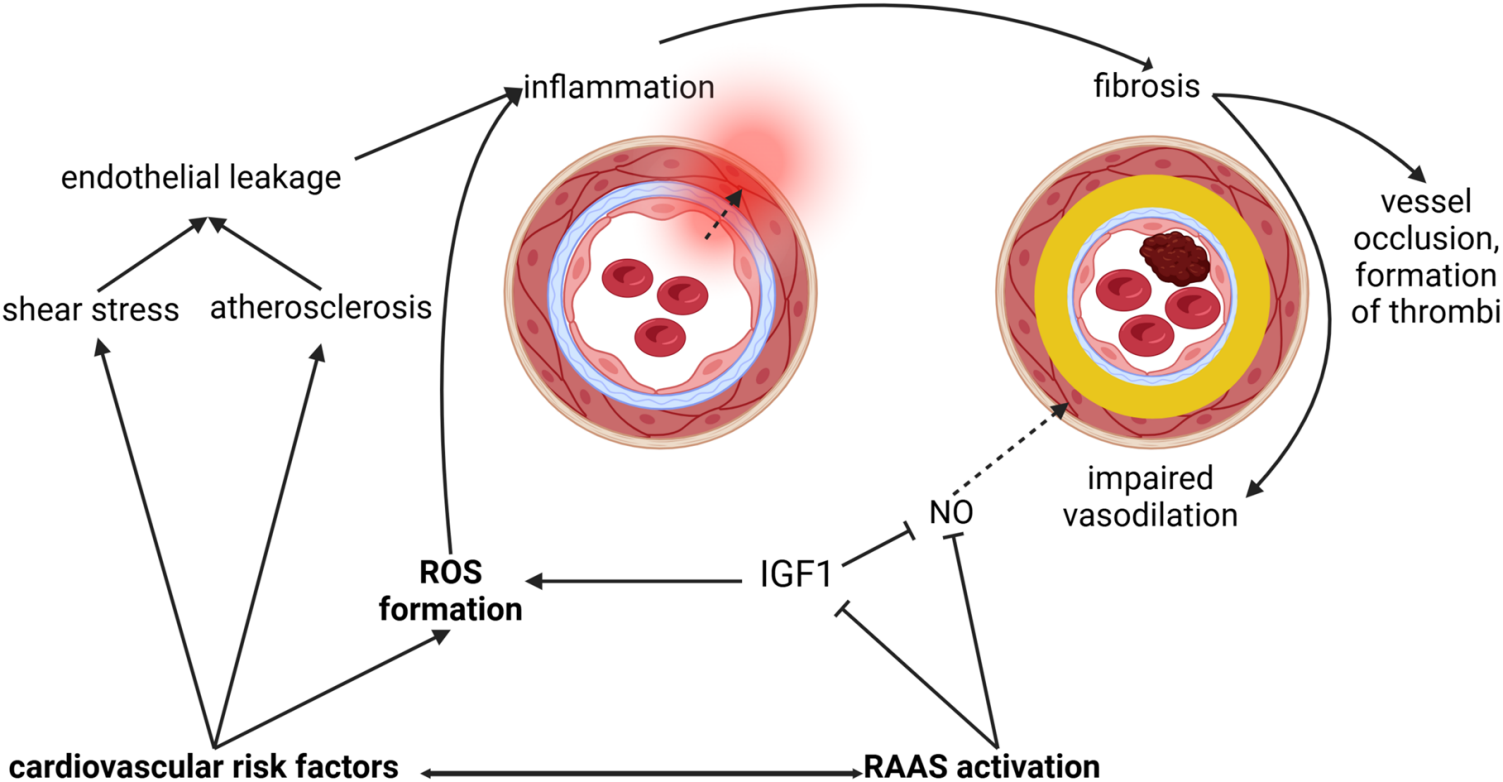
Mechanisms of endothelial dysfunction. In healthy cells, the endothelium acts as a barrier between the blood and the perivascular space and maintains vascular homeostasis. If different mechanisms such as cardiovascular risk factors, ROS formation or RAAS activation lead to endothelial dysfunction, this has various consequences. ROS formation and RAAS activation impair NO-mediated vasodilation. Cardiovascular risk factors that promote atherosclerosis or shear stress cause endothelial leakage, which allows fluids and proteins to pass through the endothelium. This leads to inflammation, which in turn results in fibrosis with thickening of the arterial wall, impairing (NO-mediated) vasodilation and causing thrombus formation. RAAS activation leads, among other actions, to a change in IGF1, which in turn leads to the formation of ROS and impaired NO-mediated vasodilatation. A vicious circle. *NO* nitric oxide, *IGF1* insulin-like growth factor-1, *ROS* reactive oxygen species, *RAAS* renin angiotensin aldosterone system, *ACE* angiotensin-converting enzyme Created in BioRender.com [17]

One mechanism that causes endothelial dysfunction is the activation of the RAAS. This has been shown to lead to changes in the insulin/insulin-like-growth factor-1 (IGF1) signaling pathways, which in turn impairs NO production and leads to ROS formation (Fig. 2) [16].

### Measurement of endothelial dysfunction

Endothelial dysfunction can be measured by various serum biomarkers as well as surrogate parameters using ultrasound.

Established biomarkers of endothelial dysfunction include cell adhesion molecules such as integrins, cadherins and selectins, which mediate the interaction between blood cells and endothelial cells and are activated during inflammation, e.g. to promote leucocyte trafficking [1, 18]. Other biomarkers are homocysteine, von Willebrand factor (vWF), interleukin-8, interleukin-18 and IGF1 deficiency [19–22]. Homocysteine interferes with NO production and causes ROS formation [23]. P-selectin, vWF and interleukin-8 are known to recruit leucocytes and platelets to the endothelium upon vascular injury [18, 19]. Interleukin-18 augments the production of inflammatory cytokines and cellular adhesion molecules [20]. In addition to other endothelial effects, IGF1 produces the vasodilator NO [21].

C-reactive protein (CRP), an inflammatory marker, has also been linked to endothelial dysfunction and cardiovascular disease risk [4]. CRP is known to be acutely and chronically elevated in ischemic stroke patients and is associated with poorer outcome. In animal models, CRP led to BBB dysfunction [1]. Interestingly, CRP was most elevated in patients with cardioembolic stroke compared to other stroke subtypes [24]. This is consistent with a suspected role of systemic inflammation in cardioembolic stroke and atrial fibrillation [25].

Apart from serum biomarkers, ultrasound techniques represent a viable method for non-invasive measurement of endothelial dysfunction. These include flow-mediated dilation (FMD), in which the diameter of a superficial artery is measured before and after inducing reactive hyperemia by inflating and deflating a cuff placed distal to the artery. In healthy individuals, reactive hyperemia would lead to shear stress and thus dilation of the artery through the release of endothelium-derived vasoactive mediators. In individuals with endothelial dysfunction, dilation is less. The result is the FMD, which indicates the percentage change in vessel diameter before and after reactive hyperemia [1]. This parameter correlates with cardiovascular risk, coronary heart disease and cardiovascular events. Another ultrasound parameter is pulse wave velocity (PWV), a marker for artery stiffness and as such mechanically linked to endothelial dysfunction [1]. Recently, transcranial color-coded doppler with breath holding techniques has been used to examine cerebral vasoreactivity as a surrogate parameter for endothelial function [26].

As these ultrasound measures are interrater-dependent, another method was developed in which peripheral arterial tone (PAT) is determined using finger plethysmography: Pneumatic plethysmographic cuffs are applied to the index and middle fingers and continuously measure the pulsatile arterial blood volume in the small arteries. Measurements are taken before and after reactive hyperemia through a cuff, similarly to FMD. The “reactive hyperemia PAT index” is quantified (the quotient of the pre and post measurements) and has shown good reproducibility, low interrater variability due to automatized scoring by software and good correlation with cardiovascular disease [27]. Another rater-independent method is MRI vessel wall imaging which has been shown to be able to evaluate endothelial shear stress and vessel wall stiffness which, however, is associated with high costs [28].

All the parameters described so far are not collected in routine clinical practice due to cost or effort. This led to the development of the endothelial activation and stress index (EASIX), which consists of three laboratory parameters that are included in most admission laboratories: creatinine, lactate dehydrogenase (LDH), and platelets and is calculated as follows: creatinine [mg/dL] * LDH [U/L]/platelet count [10^9^/L] [29]. The relationship between these three parameters and endothelial dysfunction was established by Luft et al.: The association of creatinine, LDH and platelets with thrombotic microangiopathy, a severe complication after allogeneic stem cell transplantation (alloSCT) caused by endothelial dysfunction, was previously known [30]. It is believed that these values reflect the pathophysiological response of the organism to endothelial dysfunction, rendering EASIX a rather stable value compared to the heterogeneous endothelial function explained above: Creatinine reflects renal damage, which is often caused by endothelial dysfunction, LDH production is increased in endothelial dysfunction, and a damaged endothelium leads to platelet activation and thus platelet consumption [31, 32]. Based on these considerations, a more general correlation with endothelial dysfunction was hypothesized, which was confirmed by comparison with various already known markers of endothelial damage (such as interleukin-8, interleukin-18, IGF1) [30, 33]. EASIX correlates with various cardiovascular morbidities [34] and predicts mortality in patients with coronary artery disease and in patients with acute graft-versus-host-disease (aGVHD) after alloSCT, among others [29, 35]. Suitably, a statin-based endothelial prophylaxis improved overall survival after aGVHD and alloSCT in patients with intermediate EASIX values [36].

### Endothelial dysfunction in stroke

Stroke is associated with systemic microvascular abnormalities, such as retinal microvascular abnormalities [37, 38]. Different vascular biomarkers of endothelial dysfunction have been found to be altered in acute ischemic stroke, including homocysteine, vWF, E-selectin, and intercellular adhesion molecule-1 (ICAM) [1, 22]. Plasma vWF was significantly higher in lacunar stroke than in non-stroke controls and significantly lower in lacunar stroke than in non-lacunar stroke. For other biomarkers, a robust evidence for a significant difference between lacunar and non-lacunar stroke could not be shown [1, 22]. However, biomarkers are expressed differently in distinct types of endothelium (e.g., different expression of biomarkers in capillary endothelium/arteriole endothelium/large artery endothelium), which could mask a possible difference between lacunar and non-lacunar stroke due to vessel size [5]. Therefore, a tissue-independent marker reflecting the organism’s response to endothelial dysfunction would be helpful.

Overall, it is important to keep in mind that the etiologies of ischemic stroke are numerous. In addition to the common etiologies of ischemic stroke (e.g., cardioembolic, micro- or macroangiopathic), there are ischemic stroke subtypes of unusual etiology (e.g., primary inflammatory vascular disease, infection, hematologic disorders) that imply substantially different management and treatment [39]. Not only ischemic strokes but also hemorrhagic strokes may be associated with endothelial dysfunction. For example, lacunar syndromes may also be caused by small intracerebral hemorrhages [40]. Most research to date has focused on endothelial dysfunction in ischemic stroke, although vWF levels were found to be elevated in a cohort of both ischemic and hemorrhagic stroke patients [41]. More research is needed on the topic of endothelial dysfunction in hemorrhagic stroke.

In ischemic stroke patients, overall FMD was lower and PWV was higher than in healthy controls. Those with a low FMD (< 4,5%) had a significantly worse functional outcome than those with a higher FMD, further indicating a role of endothelial dysfunction in ischemic stroke. A significant difference between different subtypes of ischemic stroke could not be shown [1].

A recent study investigated endothelial dysfunction and parenchymal hematoma after endovascular thrombectomy (EVT) in ischemic stroke. A significant correlation between a sum score of different markers of endothelial dysfunction and the risk of parenchymal hematoma after EVT was found [42]. A correlation between endothelial dysfunction and parenchymal hematoma or other outcome parameters after intravenous systemic thrombolytic therapy (IVT) has not yet been investigated.

### Drugs targeting endothelial dysfunction

Since evidence for the role of endothelial dysfunction in the pathogenesis and outcome of ischemic stroke was shown, research has been conducted on the pharmaceutical modulation of endothelial dysfunction in ischemic stroke.

Some of the medications studied have previously been approved for other purposes, such as angiotensin-converting enzyme (ACE) inhibitors for the treatment of hypertension or HMG-CoA reductase inhibitors (statins) for the treatment of high cholesterol. Both appear to have an endothelial effect beyond their primarily detected actions [43, 44]. E.g., statins are known to increase NO production and produce anti-inflammatory and anti-thrombotic agents [4]. This is supported by evidence that abrupt discontinuation of statin use in acute ischemic stroke patients leads to endothelial dysfunction and poorer neurological outcome [45]. Mevastatin was shown to decrease infarct volume, lead to lower neurological deficits while increasing levels of endothelial NO synthetase in a middle cerebral artery occlusion mice model when administered prophylactically [44]. ACE inhibitors might be beneficial due to the presumed mechanism of action of RAAS activation in endothelial dysfunction explained above, impairing NO production and conveying ROS formation through IGF1 signaling [16]. However, there have not yet been many studies examining the effect of ACE inhibitors on endothelial dysfunction in cardiovascular disease. One larger study from 2000, though, showed a significant reduction in cardiovascular events in people with a high cardiovascular risk but without reduced ejection fraction and without necessary hypertension who were treated with an ACE inhibitor [46].

Activation of the RAAS is thought to eventually lead to the upregulation of matrix metalloproteinases (MMPs), which degrade proteins of the extracellular matrix and may play a role in remodeling the endothelial environment [4, 47]. Inhibitors of certain MMPs tend to reduce infarct volume in animal models of ischemic stroke. Both doxycycline and statins inhibit MMPs [4, 47].

Some medications have only been investigated experimentally, such as the serine racemase inhibitor phenazine methosulfate and refined *Qing Kai Ling*, a preparation from traditional Chinese medicine. The former has been shown to improve cerebral blood flow (CBF) in mice after occlusion of the middle cerebral artery (MCA) [48], and the latter resulted in a reduction in ischemic infarct size as well as a reduction in neurological deficit in rats with MCA occlusion [49]. It is hypothesized that both drugs exert their effects by enhancing endothelial NO synthase (eNOS), thereby increasing NO levels and facilitating vasodilation [48, 49].

NO availability is also targeted by isosorbide mononitrate (ISMN), an NO donor that was studied in a randomized phase II trial together with cilostazol, a PDE-3 inhibitor that enhances the prostacyclin pathway and thereby has both a vasodilative and antiplatelet function [50]. ISMN reduced the incidence of recurrent stroke and cognitive disorders, and together with cilostazol, it reduced adverse vascular outcomes and dependency. A consecutive phase III study is planned [50]. Cilostazol alone has been shown to improve endothelial function better than aspirin in patients with acute ischemic stroke [51].

Serotonin reuptake inhibitors (SSRI) are another group of drugs that have been studied for their effect on endothelial dysfunction. In a systematic review from 2022, patients taking SSRI showed higher FMD than patients without SSRI, indicating better endothelial function [52]. In a small sample, serum biomarkers for endothelial dysfunction were shown to be higher before starting treatment with the SSRI escitalopram and to decrease after starting treatment [53], suggesting an improvement in endothelial function with the medication. An activation of the endothelium-dependent hyperpolarizing pathway (improving vasodilation) is considered to be the underlying mechanism [54]. To date, there are no studies that demonstrate an improvement in the outcome of ischemic stroke with SSRI medication.

Endothelial progenitor cells (EPCs) represent a new and experimental treatment option for endothelial dysfunction. These are endothelial cell-derived stem cells from the bone marrow that are activated by endothelial injury or ischemic tissue to help repair endothelial cells [55]. In animal studies, EPCs showed an effect in increasing blood flow in ischemic areas through revascularization and reducing ischemic damage in mice with MCA occlusion [4, 55]. EPCs can be isolated from umbilical cord blood and bone marrow; their effects can be enhanced by ACE inhibitors, statins, and erythropoietin, among others [4, 55]. A feared complication associated with cerebral ischemia is the development of cerebral edema after EPC-stimulating treatment [55]. Autologous EPCs have been tested in phase I and II studies in patients with ischemic stroke and showed a favorable risk profile [56]. A phase I study investigating the safety and preliminary efficacy of allogeneic EPCs in acute ischemic stroke is planned (ClinicalTrials.gov identifier NCT05993884).

**Fig. 3:**
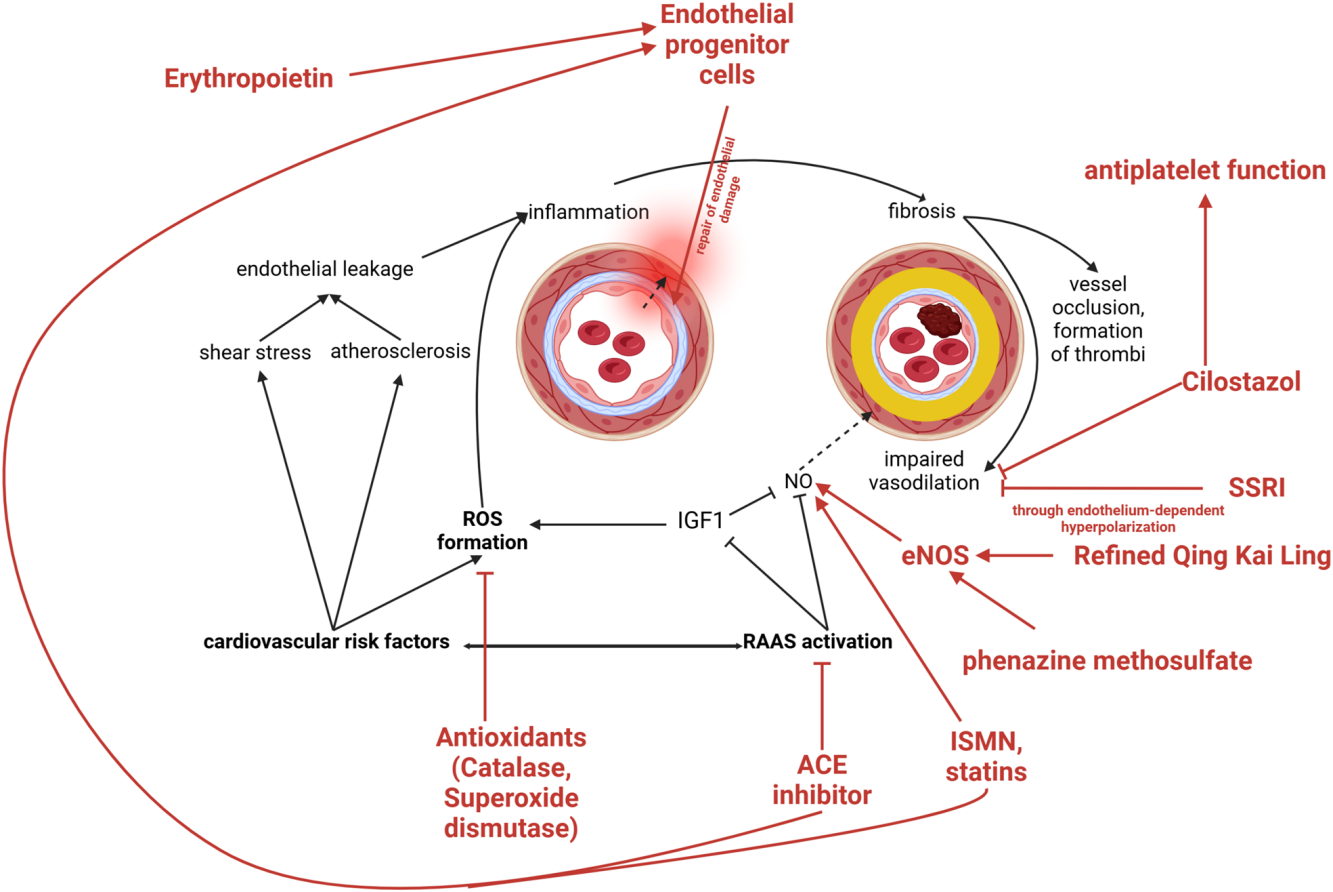
Drugs under research targeting endothelial dysfunction. The mechanisms targeted by drugs for endothelial dysfunction are complex. Many of them are directed towards NO and thereby mediating vasodilation, upregulating its presence or inhibiting its inhibitors. ROS formation, repair of endothelial damage and antiplatelet function are also addressed. *SSRI* selective serotonin uptake inhibitor, *NO* nitric oxide, *eNOS* endothelial, NO synthase, *IGF1* insulin-like growth factor-1, *ROS* reactive oxygen species, *RAAS* renin angiotensin aldosterone system, *ACE* angiotensin-converting enzyme, *ISMN* isosorbide mononitrate. Created in BioRender.com [59]

In terms of targeted therapies, efforts have been made to target endothelial cells using monoclonal antibodies directed at molecules exposed on activated endothelium (such as selectins or adhesion molecules), thereby delivering, e.g., antioxidant medication such as superoxide dismutase or catalase to the endothelium. In animal studies, these therapies have shown efficacy, particularly in lung disorders (probably owing to better bioavailability after intravenous application), but also in preserving the integrity of the BBB and improving neurological outcomes in traumatic brain injury (Fig. 3; Tables 1 and 2) [57, 58].

**Table 1:** Animal studies and clinical trials of drugs targeting endothelial dysfunction in stroke

Authors of the study and year	Drug	Study design	Number of patients/animals	Follow-up	Primary end point	Side effects	Results
**Clinical trials**							
Fang et al. 2019 [56]	Autologous EPCs after acute cerebral infarction of the middle cerebral artery territory	Two‐center, single‐blinded, randomized, parallel, placebo‐controlled	18	4 years	Safety	No severe adverse events in the EPC group	No safety concerns, outcome-wise not inferior to standard treatment (e.g., NIHSS after 3 and 48 months)
Lee et al. 2017 [51]	Cilostazol in acute cerebral ischemia patients	Double-blind placebo-controlled trial	80	90 days	FMD and L-arginine	Headache	FMD increase, L-arginine decrease
Wardlaw et al. 2023 [50]	ISMN and cilostazol in patients with cerebral small vessel disease	Investigator-initiated, open-label, blinded end-point, randomized clinical trial with a 2 × 2 factorial design	363	12 months	Recruitment feasibility and retention (primary outcome), safety, recurrent stroke, efficacy, dependency and others (secondary outcomes)	Headache, palpitations, dizziness, loose stools, nausea, bleeding, dyspepsia, bruising, and falls	Study feasible, drugs well tolerated and safe, may reduce recurrent stroke, dependence, and cognitive impairment after lacunar stroke
**Animal Studies**							
Amin-Hanjani et al. 2001 [44]	Mevastatin (prophylactic intake) in a MCAO model	Animal study (mice)	9–12	24 h	Infarction volume, neurological deficit, cerebral blood flow, eNOS mRNA and protein levels	NA	Infarction volume reduced, neurological deficits improved, levels of eNOS mRNA and protein increased
Hua et al. 2008 [49]	Refined Qing Kai Ling in a MCAO model	Animal study (rats)	6	72 h	Infarction volume, neurological deficit, activity/expression level of eNOS protein	NA	Infarction volume smaller, neurological deficits lower, eNOS activity and expression higher in rats treated with the study medication
Watanabe et al. 2016 [48]	Serine racemase inhibition in a MCAO model	Animal study (mice)	3–12	48 h	Cerebral blood flow, infarct volume, neurological deficit	NA	Improved cerebral blood flow, reduced infarct volume, improved neurological deficit

*MCAO* middle cerebral artery occlusion, *EPCs* endothelial progenitor cells, *ISMN* isosorbide mono nitrate, *NIHSS* National Institute of Health Stroke Scale, *FMD* flow-mediated dilation, *eNOS* endothelial nitric oxide synthetase

**Table 2:** Main results of the most relevant papers analyzed in this review in the order of their date of publication

Authors and year of publication	Summary of main results
Amin-Hanjani et al. 2001 [44]	Mevastatin as an activator of endothelial NO synthetase decreases infarct volume and reduces neurological deficits in a middle cerebral artery occlusion mouse model
Hua et al. 2008 [49]	Refined Qing Kai Ling reduces infarct size, reduces neurological deficit and enhances endothelial nitric oxide synthase activity in rats with middle cerebral artery occlusion
Doubal et al. 2009 [37]	Stroke is associated with systemic (e.g. retinal) microvascular abnormalities
Wiseman et al. 2013 [22] / Tuttolomondo et al. 2020 [1]	Different vascular biomarkers of endothelial dysfunction are altered in acute ischemic stroke
Kurzepa et al. 2014 [47]	Inhibitors of matrix metalloproteinases decrease the infarct volume in animal models of ischemic stroke
Watanabe et al. 2016 [48]	Improved cerebral blood flow in mice after occlusion of the middle cerebral artery when treated with serine racemase inhibitor phenazine methosulfate
Fang et al. 2019 [56]	Autologous endothelial progenitor cells show no safety concerns and are non-inferior to standard-of-care treatment
Tuttolomondo et al. 2020 [1]	Flow-mediated dilation and pulse wave velocity as markers of endothelial dysfunction are lower, respectively, higher in ischemic stroke patients
Delialis et al. 2022 [52]	Selective serotonin reuptake inhibitors significantly increase flow-mediated dilation
Lu et al. 2022 [55]	Endothelial progenitor cells increase blood flow and reduce ischemic damage in ischemic areas in mice with middle cerebral artery occlusion
Zhang et al. 2023 [42]	Endothelial dysfunction correlates with risk of parenchymal hematoma after endovascular therapy for ischemic stroke
Wardlaw et al. 2023 [50]	Isosorbide mono nitrate and cilostazol together reduce adverse vascular outcomes and dependency in cerebral small vessel disease

### Strengths and limitations

This review aimed to assess the role of endothelial dysfunction in acute ischemic stroke and therapies currently under investigation for the treatment of endothelial dysfunction. We used a specified search strategy and screened the references of the articles selected. As a limitation, this is not a systematic review, and only English- and German-language papers were included.

## Conclusion

The role of endothelial dysfunction in acute ischemic stroke is an area of ongoing research. The fact that there is a correlation between the two clinical problems has been demonstrated by several studies and there are plausible pathophysiological rationales for this.

However, there is a lack of routine clinical use for most of the surrogate endothelial dysfunction parameters. To close this gap in clinical stroke treatment we propose to investigate the relation of biomarkers that are easier to obtain and outcome in acute ischemic stroke in large cohorts. In addition, it would be of interest to examine the role of endothelial dysfunction on complications and overall outcome after the two available acute therapies for ischemic stroke, IVT and EVT, as started by Zhang and colleagues [42]. This might promote a more personalized approach in decision-making for or against these therapies. Regarding the differences in endothelial structure and function, it may be informative to collect samples from the specific vascular beds (e.g., cerebral vessels during EVT). The role of endothelial dysfunction in different etiologies of ischemic stroke needs to be further investigated, as the studies performed so far are contradictory. One could well imagine a difference in endothelial function between cardioembolic, macroangiopathic and microangiopathic causes of stroke.

With regards to therapies targeting endothelial dysfunction, most of the substances studied are still at a very experimental stage of research. However, early clinical trials are being conducted in which, e.g., endothelial progenitor cells are investigated. More clinical trials are warranted to translate promising approaches from animal studies to human clinical trials, e.g., serine racemase inhibition, or to investigate agents that have been studied with regard to endothelial dysfunction but not in the context of stroke, e.g., SSRIs. The development of new drugs specifically targeting endothelial dysfunction may also be beneficial. However, in terms of a critical use of resources, it would be prudent to first investigate in prospective clinical trials the beneficial effects of drugs already widely used in the clinic on endothelial dysfunction in stroke.
